# EPViz: A flexible and lightweight visualizer to facilitate predictive modeling for multi-channel EEG

**DOI:** 10.1371/journal.pone.0282268

**Published:** 2023-02-27

**Authors:** Danielle Currey, Jeff Craley, David Hsu, Raheel Ahmed, Archana Venkataraman

**Affiliations:** 1 Department of Computer Science, Johns Hopkins University, Baltimore, MD, United States of America; 2 Department of Electrical and Computer Engineering, Johns Hopkins University, Baltimore, MD, United States of America; 3 Department of Neurology, University of Wisconsin Madison, Madison, WI, United States of America; 4 Department of Neurosurgery, University of Wisconsin Madison, Madison, WI, United States of America; 5 Department of Electrical and Computer Engineering, Boston University, Boston, MA, United States of America; Kuwait College of Science and Technology, KUWAIT

## Abstract

Scalp Electroencephalography (EEG) is one of the most popular noninvasive modalities for studying real-time neural phenomena. While traditional EEG studies have focused on identifying group-level statistical effects, the rise of machine learning has prompted a shift in computational neuroscience towards spatio-temporal predictive analyses. We introduce a novel open-source viewer, the EEG Prediction Visualizer (EPViz), to aid researchers in developing, validating, and reporting their predictive modeling outputs. EPViz is a lightweight and standalone software package developed in Python. Beyond viewing and manipulating the EEG data, EPViz allows researchers to load a PyTorch deep learning model, apply it to EEG features, and overlay the output channel-wise or subject-level temporal predictions on top of the original time series. These results can be saved as high-resolution images for use in manuscripts and presentations. EPViz also provides valuable tools for clinician-scientists, including spectrum visualization, computation of basic data statistics, and annotation editing. Finally, we have included a built-in EDF anonymization module to facilitate sharing of clinical data. Taken together, EPViz fills a much needed gap in EEG visualization. Our user-friendly interface and rich collection of features may also help to promote collaboration between engineers and clinicians.

## Introduction

Scalp electroencephalography (EEG) has long been used as a window into the complex inner-workings of the human brain. Formally, EEG measures the effects of postsynaptic currents in the brain and provides real-time information about neural activity [1, 2]. Its cost-effectiveness and relative ease of acquisition has made EEG ubiquitous in both research and clinical practice. To a large extent, traditional EEG analysis has focused on group-level effects. Broadly, these studies extract quantitative features from the EEG data and use statistical testing either to identify significant differences between groups or to compute the explained variance with respect to some external measure. Common features include the amplitude and timing of evoked response potentials (ERPs) [3, 4], spectral power across the standard EEG frequency bands [5–7], quantitative metrics of the brain network organization [8–10], and spatial arrangement of ICA components [4, 11]. One commonality across these methods is that they draw “static” conclusions at the level of an EEG channel or a brain network. Hence, visualization of these findings is straightforward.

The rise of machine learning has spurred new directions in computational electrophysiology focused on time-varying and patient-specific predictive analyses. This paradigm shift has been accelerated by deep learning and platforms, such as PyTorch and TensorFlow, which make such techniques readily available to the research community. Two common application domains are epilepsy monitoring and brain computer interface (BCI) systems. Much of the work in epilepsy focuses on the problem of seizure detection. This setting is often cast as a binary classification problem, where the goal is to classify whether short windows (1-10 sec) of multi-channel EEG correspond to baseline or seizure activity [12–14]. The methods range from traditional machine learning algorithms applied to hand-crafted features, such as wavelet coefficients [5, 15–21], spectral power [6, 7, 22–26], and non-linear measures [5, 17, 20, 27–31], to end-to-end deep neural networks based on convolutional and recurrent architectures [32–44]. Recent work in epilepsy has pivoted towards localizing the seizure onset from EEG, which adds a spatial component to the temporal predictions [23, 45, 46]. On the other hand, BCI systems try to decode user intent based on the EEG signals in order to control the environment [47]. One approach detects sensorimotor rhythms generated by motor imagery [48, 49], typically by evaluating the EEG frequency content in the C3 and C4 electrodes [50]. Similarly, steady state visually evoked potentials measure stable responses to flickering visual stimuli [51]. These potentials are observed in the occipital lobe and can be detected using methods such as filterbank analysis and canonical correlation analysis [52].

Software packages for EEG can be divided into two categories. The first category focuses on specific analytical techniques, with the visualization options for each package highly targeted towards the method under consideration. Examples include EEGLab [53, 54], which is geared towards ERP analysis, EEGNet [55], which emphasizes brain connectivity and network analyses, and BrainStorm [56], which tries to link multimodal information in a common reference space. While these software packages represent seminal contributions to the field, none of them are geared towards viewing the results of time-varying and spatially-varying predictive analyses. The second category of software includes EEG viewers that display and manipulate the raw time series data. The most popular viewer is EDFBrowser [57], which provides a wide range of preprocessing, display, and annotation functionalities. While EDFBrowser is and will remain a valuable resource to the community, it has some notable limitations. For example, the large number of tools makes the interface clunky and difficult to navigate. In addition, EDF Browser does not have native support for visualizing model predictions, a need that is growing in popularity with machine learning analyses.

In this paper, we introduce the EEG Prediction Visualizer (EPViz), a lightweight and flexible EEG viewer that complements existing software resources in the field. EPViz is targeted towards machine learning applications and is built around four core functionalities: (1) displaying and manipulating the multi-channel EEG time series, (2) running PyTorch deep learning models on the data, (3) overlaying channel-wise and time-varying predictions on top of the EEG time series, and (4) saving high-quality images of the results. In addition, EPViz includes basic preprocessing operations, spectral feature extraction, and annotation editing. Finally, EPViz has a built-in anonymizer to facilitate sharing of clinical EEG data between clinicians and engineers. EPViz is freely available for download at https://engineering.jhu.edu/nsa/links/.

## Materials and methods

EPViz is a streamlined viewer designed for predictive modeling applications. EPViz is built using the PyQt package (5.15.4) in Python. PyQt allows for easy integration with a range of Python deep learning and machine learning libraries.

The multi-channel EEG data is plotted using the PyQtGraph package, which provides fast updating and real-time user interaction capabilities. The PyEDFlib package is used for loading EDF files, and the Matplotlib package is used for saving high-quality images. Finally, the MNE package [58] is used to generate a 2-D topographic map of channel-wise model predictions on the scalp for enhanced visualization capabilities. This representation is also known as a topoplot.

### Overview of the GUI

**Fig 1:**
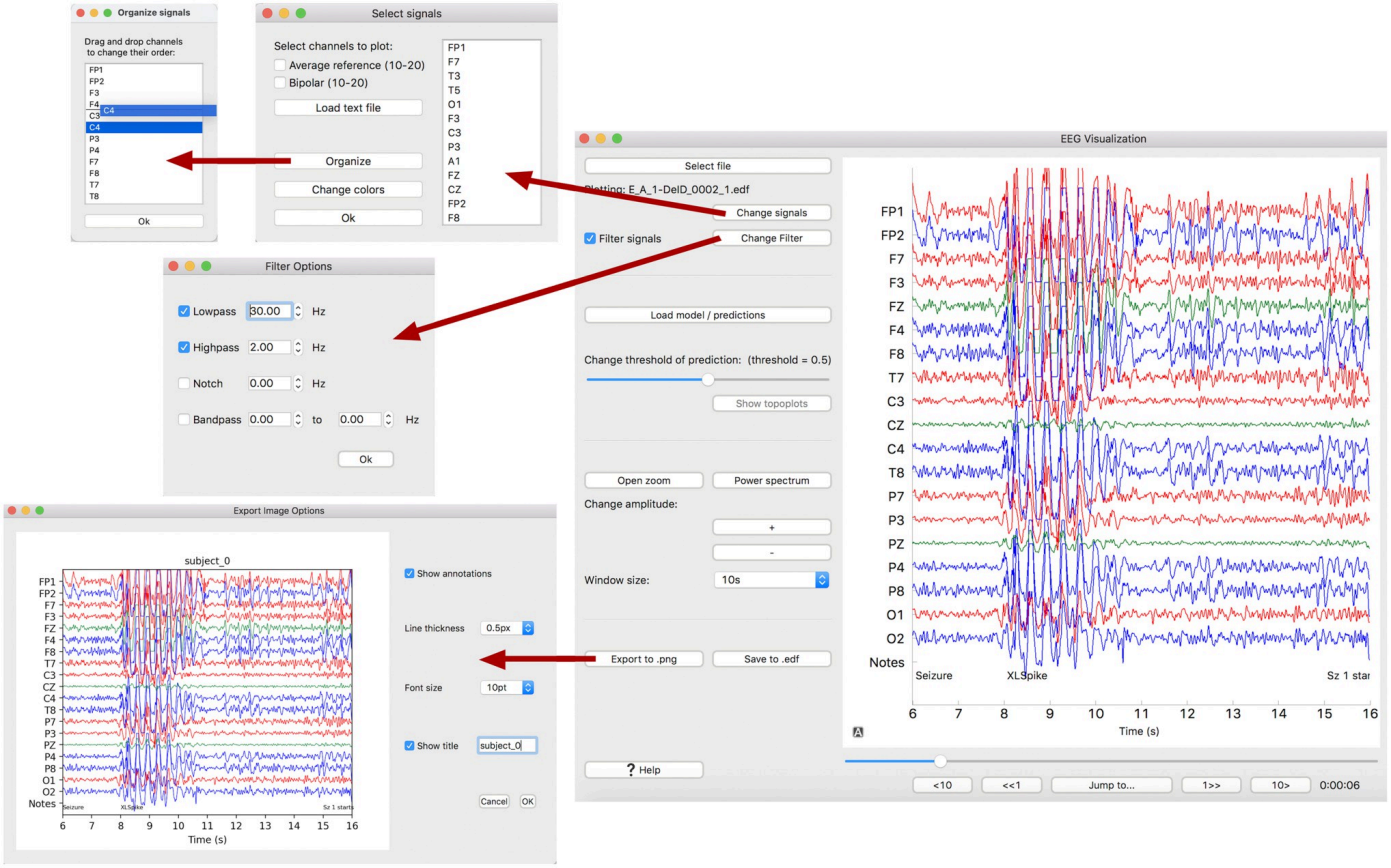
Main GUI window. The signal organization window can be used to change the order of signals (top left). This window is opened from the signal selection window. Also shown are the filtering and image saving windows. The main window includes a leftside-panel with various options and the main signal plot to the right.

Fig 1 illustrates the EPViz graphical user interface. The “Select File” button allows the user to load an EDF file containing multi-channel EEG data. The popup window asks the user to select which channels to plot. We have included the standard 10-10, 10-20 and bipolar 10-20 montages as preset selections. The user also has the option to load a custom EEG montage via a separate text file.

The EEG signals appear in the main display pane. Signals from the default montages are color-coded according to hemisphere (red for left, blue for right, and green for the midline). This is in contrast to EDFBrowser, which defaults to plotting all signals in black. Users can change the ordering and number of plotted signals using the “Change Signals” button. Annotations in the EDF files are plotted as “Notes” at the bottom of the display pane. These are particularly relevant for clinical EEG data. Users can vary the time scale of the plot (1, 5, 10, 20, 25, 30, or 45 seconds) using the “Change Window Size” button. Likewise, they can change the intensity scale via the “Change Amplitude” button. Finally, the “Open Zoom” button allows the user to zoom in on a selected region of the plotting window.

EPViz includes basic filtering operations. The high- and low-pass parameters, implemented using the SciPy library, can be set in the “Change Filter” pop-up. To allow for real-time updating, only the region shown on the screen is filtered. These filtering operations mimic those used in epilepsy and BCI applications. More complex preprocessing, such as ICA, should be done offline prior to loading the file into EPViz.

### Obtaining and displaying temporal predictions

EPViz supports two types of predictions. The first is a continuous value between [0, 1], corresponding a soft binary assignment. By default, EPViz assumes that “0” is the baseline condition and “1” is the condition of interest. The second is a categorical assignment into one of *K* classes plus a default class of “0” again denoting a baseline condition outside of the main assignments.

The user can load predictions in one of two ways. The first method is via an auxiliary file. The file should either contain a single row, corresponding to a subject-level prediction for each time point, or contain the same number of rows as plotted EEG channels, corresponding to a channel-wise prediction for each time point. The second method is by loading a pre-trained PyTorch model and running it directly on loaded data. Here, the PyTorch model should generate an output that is an integer modulo the number of samples in the signal. This format accounts for models that generate window-wise predictions across short (e.g., 1-10 sec) snippets of the full EEG recordings.

#### Figure generation and export

**Fig 2:**
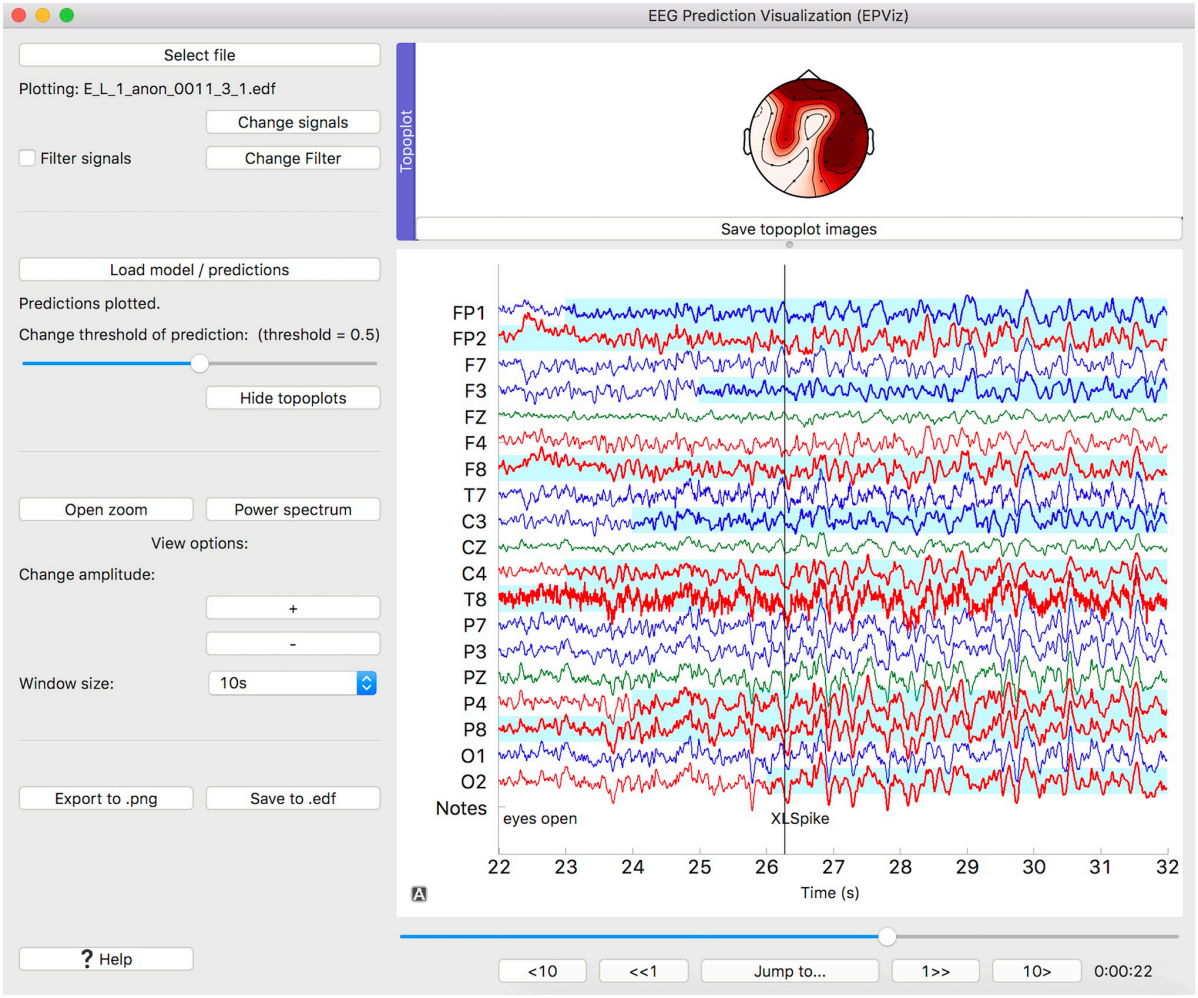
Visualizing predictions. Model predictions are shown in light cyan across the channels. Since multi-channel predictions are plotted, topoplots have been generated.

Fig 2 illustrates the model predictions in the main display pane. As seen, the predictions are overlaid in a light cyan across the appropriate channels. In the case of binary classification, the detection threshold can be swept using the built-in slider bar. This strategy allows users to identify salient features of the underlying EEG that may coincide with the predictions. Not only does this mimic clinical review of scalp EEG data, but it may facilitate interpretability of the corresponding algorithms.

Going one step further, EPViz can display multi-channel predictions on a topological scalp plot (topoplot). The topoplots are generated using the MNE plot_topomap function. The user can select the time point to display by moving a black vertical line on the main display pane. Once positioned, the topoplot is automatically updated as the user scrolls through the EEG data.

Finally, the “Save to .png” option allows the user to export a high-quality image of the main display pane. Here, the user first selects the desired options (filtering signals, overlaying predictions, adding annotations) and proceeds to an image editor. The editor allows them to change the plot title, the EEG signal thickness, and the text font size. Topoplots can be exported to an image using the “Save topoplot” button. Users can toggle subplot titles which default to the times for each topoplot. The Matplotlib package is used for exporting the * .png files.

### Other functionalities

#### Data statistics and spectrum

**Fig 3:**
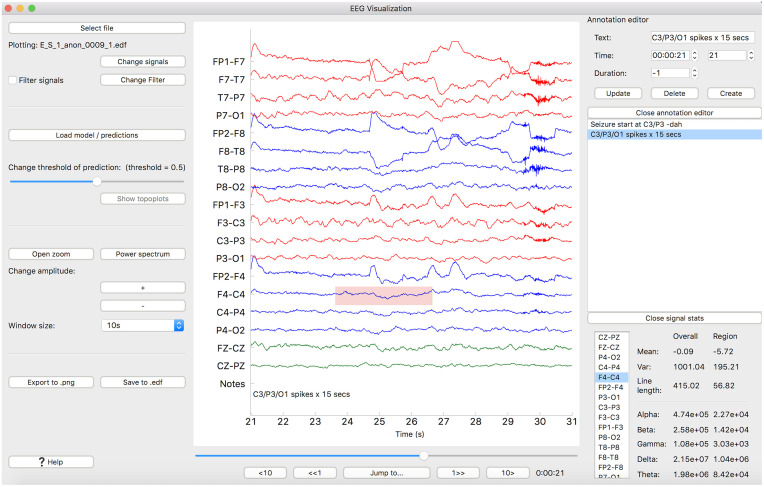
Annotation editor and statistics. The dock on the right side of the plot contains the annotation editor (top) and signal statistics (bottom). The red rectangle on the main plot can be used to select a region over which statistics will be computed.

EPViz computes and displays basic statistics of the EEG data. These include signal mean, variance, and line length. Line length is computed as the sum of distances between consecutive time points of the signal; it is a particularly useful metric in EEG analysis [29]. Beyond these time-domain features, EPViz computes the power within the standard EEG frequency bands: delta (1-4 Hz), theta (4-8 Hz), alpha (8-14 Hz), beta (14-30 Hz), and gamma (30-45 Hz). As shown in Fig 3, the user can control the channel and time interval over which the statistics are computed by moving the red rectangle.

EPViz also plots the spectrogram of a selected EEG channel. The spectrogram is extracted based on the Fast Fourier Transform magnitude. This time-frequency representation is popular in many EEG applications [6, 7, 22–26]. Users can toggle the spectrogram via the “Power Spectrum” button. EPViz computes and displays basic statistics of the EEG data. These include signal mean, variance, and line length. As its name suggests, line length is computed as the sum of distances between consecutive time points of the signal; it is a particularly useful metric in EEG analysis. Beyond these time-domain features, EPViz computes the power within the standard EEG frequency bands: delta (1-4 Hz), theta (4-8 Hz), alpha (8-14 Hz), beta (14-30 Hz), and gamma (30-45 Hz). As shown in Fig 3, the user can control the channel and time interval over which the statistics are computed by moving the red rectangle.

#### Annotation editor

The EDF file format provides a means to store text annotations linked to specific time points of the data. Annotations are often added by clinicians to flag salient events, for example during epilepsy monitoring. The are also useful in research studies to indicate the timing of different stimuli or experimental conditions. Similar to EDFBrowser, our EPViz includes tools for extracting and modifying textual annotations. Specifically, the annotations are displayed both as a list on the right of the window and in the “Notes” row of the main display pane. As seen in Fig 3, we have also included an annotation editor that lets the user both modify the text of existing annotations and add new annotations. Changes made using the annotation editor will not persist outside of EPViz unless they are saved into a new EDF file.

#### Clinical anonymization

**Fig 4:**
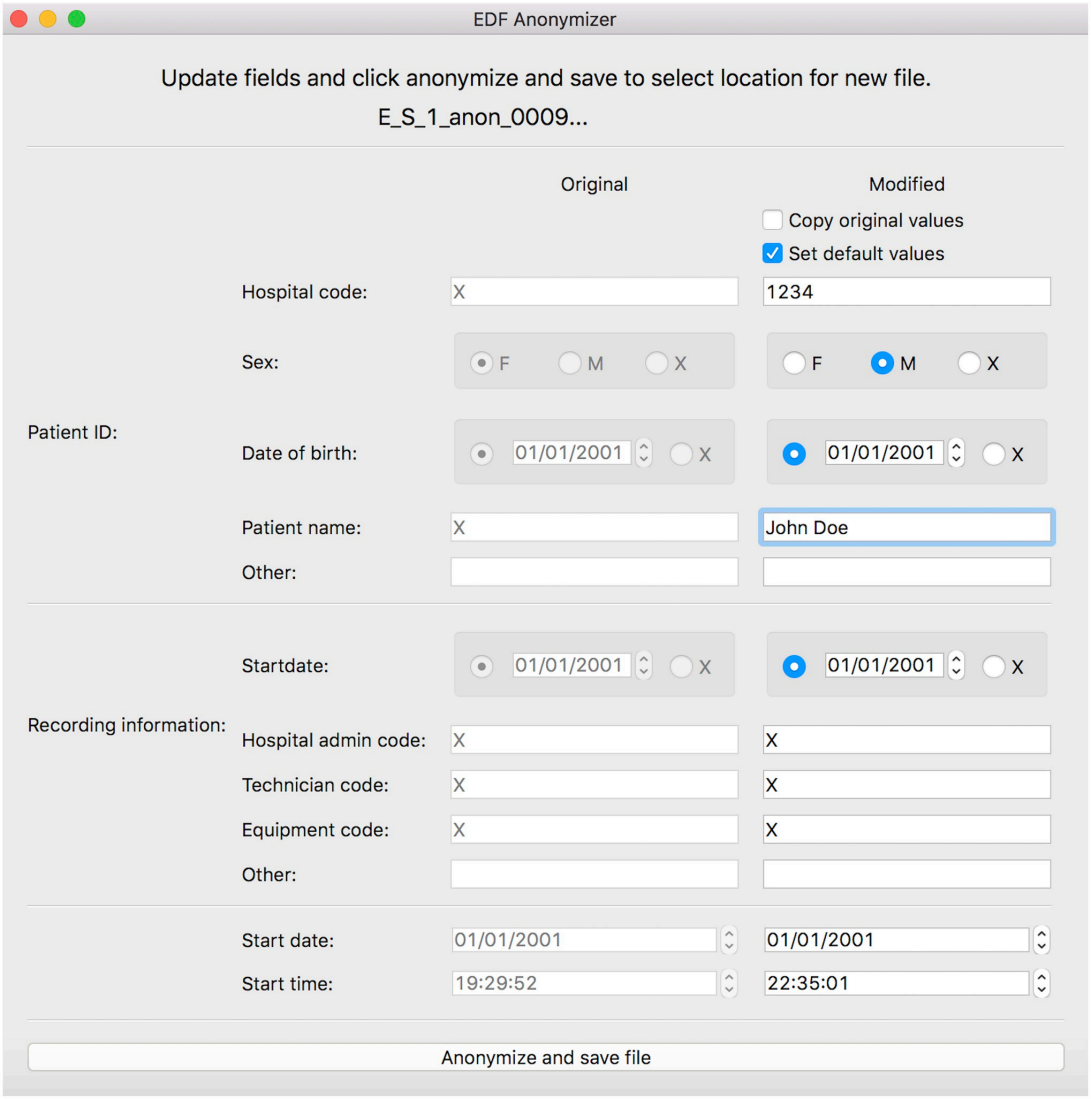
Anonymization window. This option can be used to strip identifiable information from the EDF file prior to saving.

To facilitate the sharing of clinical data, EPViz includes a built-in anonymizer to strip identifiable information from the EDF file prior to it being saved. Fig 4 shows the annonymization window. Here, users can opt for the default setting, which removes names and dates from the EDF header, or they can selectively edit each field themselves. The Python code underlying the anonymizer has been verified by the University of Wisconsin (UW) Madison Institutional Review Board and is currently being used for data sharing between UW Madison and Johns Hopkins University.

For convenience, the EEG data and the overlaid model predictions can be saved into a single EDF file. This file can easily be re-loaded into the EPViz for further analysis and visualization. Logistically, the model predictions are stored as a new data channel and should not interfere with other EEG software packages.

#### Command line options

We have provided command-line support for figure generation of the main display pane (EEG signals) and for data anonymization. These command-line option can be integrated into batch processing pipelines and are particularly useful for comparing different predictive modeling outputs. In summary, EPViz is built so that people with and without technical expertise are able to easily interact with its tools.

### Development and release

#### Software testing procedures

To ensure a smooth user experience, we have added unit testing for EPViz. Our tests cover the main functionalities of the visualizer (e.g., loading, plotting, manipulating, and saving data/images) with extensive coverage of the corresponding source files. Exceptions included purely UI functionalities, which require constant user interaction. Table 1 shows the code coverage of our unit tests. We have also created a GitHub Action to run the unit tests on each pull request to encourage high-quality code integration.

**Table 1:** Unit testing code coverage for EPViz source files.

Functionality	File	Coverage (%)
Main plot	plot.py	82
	plot_utils.py	93
EDF saving	anonymizer.py	91
	saveEdf_info.py s	100
	saveEdf_options.py s	100
Filtering	filter_options.py	100
Predictions	prediction_info.py	82
	prediction_options.py	74
Preprocessing	edf_loader.py	100
Signal loading	channel_info.py	93
	channel_options.py	78
Signal statistics	signalStats_info	100
	signalStats_options	92
Spectrograms	spec_options.py	99
Image saving	saveImg_options.py	81
	saveTopoplot_options.py	93

Finally, we have used Pylint (https://pylint.pycqa.org/en/latest/) during the development process to ensure that our EPViz source code conforms to the PEP 8 style guidelines for Python.

#### Software dissemination

We have included three ways for users to download and install EPViz. First, users can clone our GitHub repository, which contains the most up-to-date version of the code. The repository includes information for developers about how to use EPViz along with test EDF files from the public Children’s Hospital of Boston (CHB) and Temple University Hospital (TUH) datasets [25, 59, 60] that can be used to explore the visualizer functionality. The GitHub repository is linked on our lab webpage: https://engineering.jhu.edu/nsa/.

Second, EPViz is available on PyPI at https://pypi.org/project/EPViz. This page provides instructions on how to install EPViz in Linux, MacOS and Windows, links to our online documentation, and a summary of features and command-line options. There is also a description of the unit tests created for EPViz and instructions for running pylint on any code modifications to ensure compatibility.

Third, EPViz can be downloaded as a standalone package for MacOS and Windows. This option is geared towards users with limited programming experience, who simply want to access the functionalities of EPViz. These packages are available for download at https://engineering.jhu.edu/nsa/.

EPViz is licensed under General Public License (GPL) 3.0.

#### Online documentation

We have created an extensive online documentation page for EPViz, which can be accessed via our lab website at: https://engineering.jhu.edu/nsa/epviz/. The documentation includes a short video of EPViz, followed by detailed information about each of the main features and tips to help users interact with the visualizer. The user can also download the test EDF files in our GitHub repository and follow along with a step-by-step demo at the end of the page. This demo reviews a common use case for EPViz including loading a file, selecting a montage, loading predictions, navigating through the signal time series, saving a figure, and anonymizing the file. These steps are also covered in the linked video.

## Real-world use case: Seizure detection

### Data

**Fig 5:**
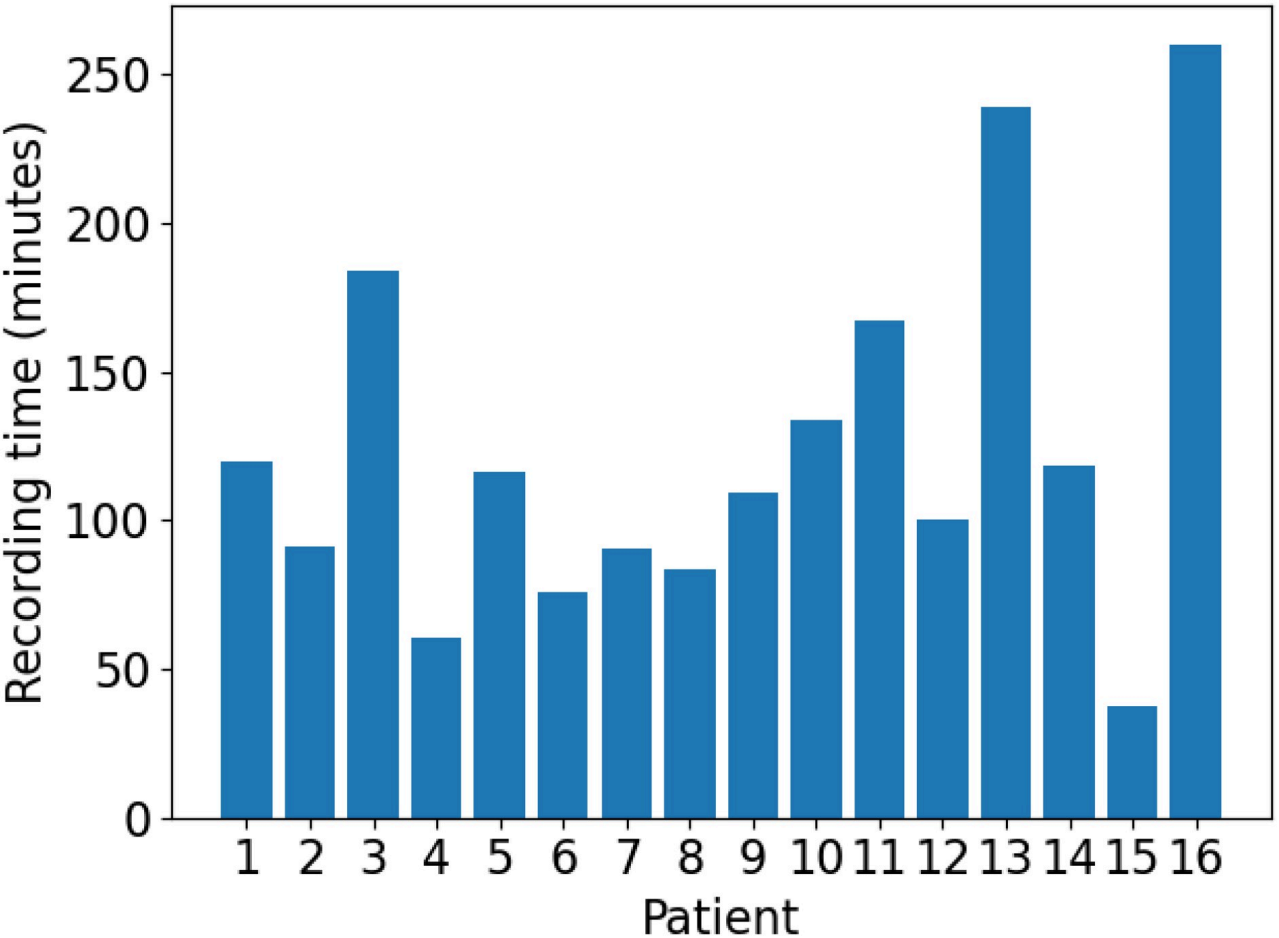
UWM dataset recording times. Total time in minutes of EEG included for each patient in the UWM dataset.

We demonstrate the real-world utility of EPViz via a seizure detection experiment. Our scalp EEG dataset was acquired by University of Wisconsin-Madison pediatric epilepsy monitoring unit. It includes 192 EEG recordings in the standard 10-20 montage [61] from 16 patients for a total of 100 seizures. There are an average of 6.25 seizures per patient (min = 1 seizure, max = 33 seizures). The total recording time is 33.1 hours with an average of 124 minutes of recorded EEG per patient, as seen in Fig 5. The EEG data was sampled at 256 Hz and resampled to 200 Hz for the purpose of analysis. The EEG was annotated for seizure onset and offset by a clinician using video-EEG.

### Experimental setup

#### Predictive models

We compare the performance of eight different methods for seizure detection drawn from recently published work. These methods encompass a range of feature extraction and machine learning techniques. The inputs to each method are one-second windows of multichannel EEG data, provided as a sequence. The outputs are window-level predictions of seizure versus baseline activity. Here, we provide a concise summary of each method and refer the reader to the citations below for additional details.

- **CNN-BLSTM**: Introduced in [32], this deep learning architecture couples a convolutional neural network (CNN) feature encoder with a recurrent bidirectional long short-term memory (BLSTM) classifier.
- **CNN-MLP**: Proposed as a baseline for the CNN-BLSTM [32], this method uses the same convolutional encoder, but replaces the BLSTM with a multi-layer perceptron that operates independently on each one-second window of the EEG.
- **Wei-CNN**: Developed by [41], this is a fully-convolutional deep learning method that uses a single CNN to obtain window-wise seizure versus baseline predictions.
- **CNN-2D**: Also used a baseline in [32], this method concatenates the FFT of the channel-wise EEG signals into a 2D matrix and operates on it like an image. This method is inspired by the works of [34, 37], which use a similar strategy.
- **MLP**-XXX: These three methods rely on hand-crafted features extracted channel-wise from the one-second windows as described in [45]. The “time” features consist of sample entropy, signal energy, line length, and largest Lyapunov exponent. The “filterbank” features consist of spectral power in different EEG frequency bands. The classification is performed by a multi-layer perceptron.
- **Kaleem-SVM**: As introduced in [18], this method operates on the combined time and filterbank features described above but uses a support vector machine (SVM) classifier instead of a deep neural network.

Training and calibration is performed according to [32]. For the CNN-BLSTM, the CNN encoder is pre-trained on individual windows for 10 epochs prior to joint training of the full architecture. The outputs for each method are averaged 20 consecutive windows to reduce noise in the final predictions. Likewise, the seizure detection thresholds for each method are independently set to allow only two minutes of false positive seizure classifications per hour *on the training data*.

Finally, we note that our objective in this study is to demonstrate how EPViz can be used to visualize the results of a real-world predictive analysis, rather than to advocate for any particular seizure detection method. Thus, we have selected models that are simple to implement and train, while still being current in the field.

#### Performance metrics

We evaluate performance at the level of one-second EEG windows and at the level of whole seizures. In the former case, we treat the window-level seizure versus baseline predictions as independent outputs and compute the Area Under the Receiver Operating Characteristic Curve (AUC-ROC) and the Area Under the Precision-Recall Curve (AUC-PR). These metrics capture the behavior when varying the detection threshold. We also compute sensitivity and specificity using the detection thresholds obtained during callibration (see previous section). In the latter (whole seizure) case, we first determine the intervals of contiguous seizure classification based on the callibrated detection thresholds. Any interval that intersect a clinician-annotated seizure is considered a true positive detection; the remaining are considered false positive detections. From here, we quantify the duration of false positive detections, the sensitivity (true positives divided by total number of seizures), and the onset latency.

#### Experimental results

**Fig 6:**
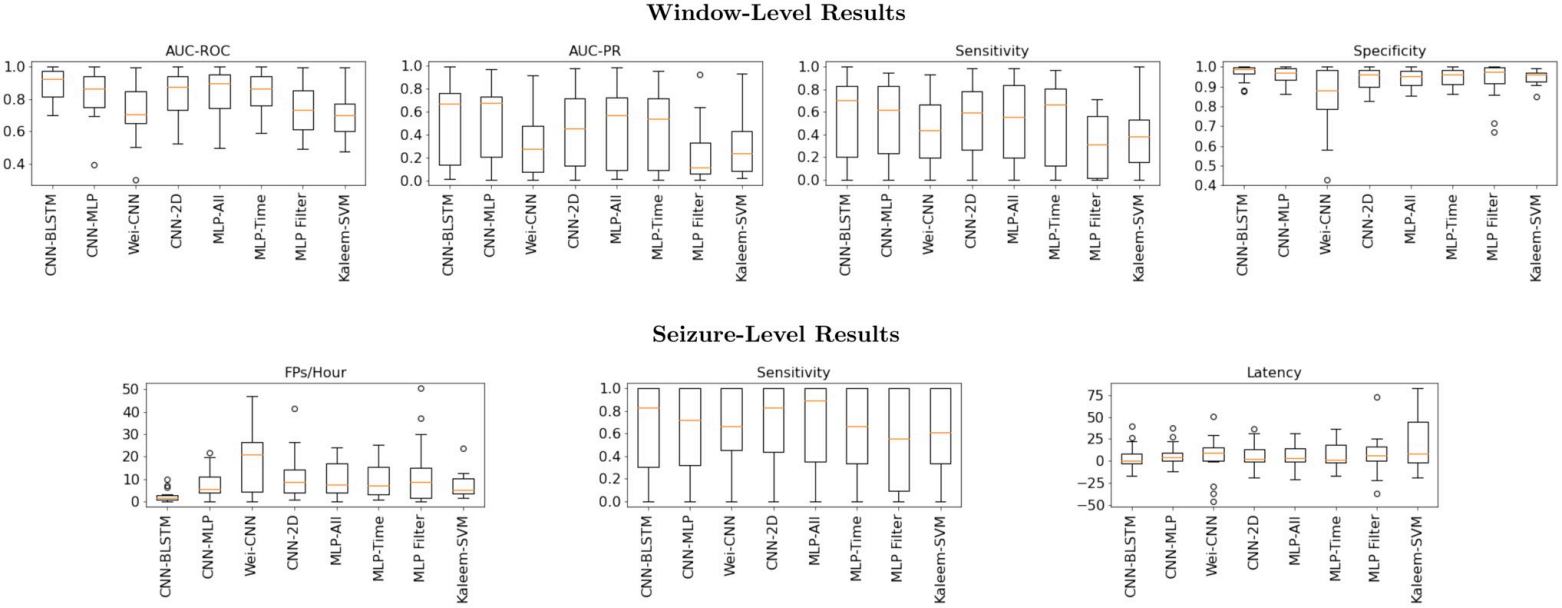
Seizure detection performance on the UWM dataset. Box plots capture the average performance for each metric across the 16 patients. The orange bar denotes the median performance, boxes correspond to the inter-quartile range, and the whiskers denote the 10th and 90th percentiles. **Top**: Window level results are calculated on one-second windows of EEG. **Bottom**: Seizure level results are calculated over the duration of the seizure period.

Fig 6 reports the seizure detection results. The box plots are constructed by first averaging the performance metrics across all seizures for a given patient and then plotting the distribution of these averages across patients. Note that the CNN-BLSTM and CNN-MLP have the best median performance at the window level, which suggests that the convolutional encoder is key to learning discriminative representations from the data. At the seizure level, while the deep learning methods achieve similar sensitivity and onset latency, the CNN-BLSTM shines with respect to low false positives. This observation indicates that (1) the calibration thresholds set during training are more likely to generalize to testing data for the CNN-BLSTM, and (2) the temporal modeling of this architecture may help to suppress less certain seizure predictions.

**Fig 7:**
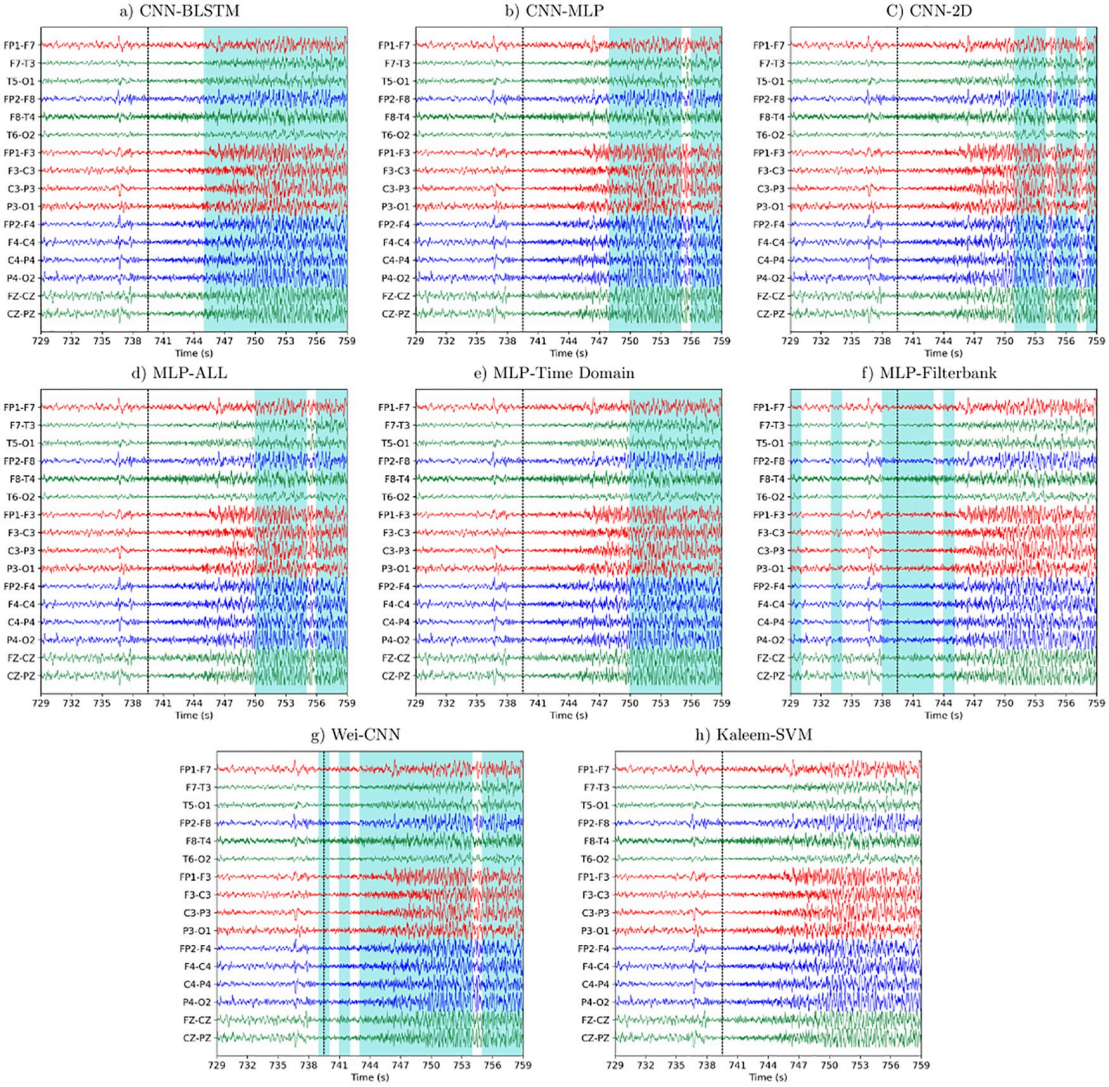
EPViz visualizations of seizure detections from each model. Detected seizures are shown superimposed in blue on the EEG. Annotated seizure onset at 739 seconds is shown by the vertical dashed line. The CNN-BLSTM shows a contiguous seizure detection with low latency while alternative models show higher latency, non-continuous detection, or misses.

Fig 7 demonstrates the utility of EPViz in comparing different methods. Here, the unsmoothed seizure detections for each model are superimposed in blue over the original EEG signal, here displayed using the longitudinal bipolar montage. Annotated seizure onset is displayed using the dashed vertical line at 739 seconds. The CNN-BLSTM makes a continuous seizure detection with low latency. With the exception of the MLP-Time Domain, CNN and MLP models make non-continuous seizure classifications, alternating between detected seizure and periods of baseline. The Kaleem-SVM misses the seizure entirely. Beyond this example, EPViz can be used to compare model behaviors during baseline activity and seizure offset. Researchers can also use these images to identify confounding features in the underlying EEG signals, then can be re-incorporated into their models for improved performance.

Finally, we note that the seizure detection performance in the UWM dataset is lower than the performance of the models reported in [32] using the CHB dataset [25, 26]. This trend may be partially attributed to the smaller sample size of the UWM dataset. In addition, the CHB dataset contains primarily generalized seizures, whereas the UWM dataset contains a more heterogeneous focal epilepsy cohort.

## Discussion

### Comparison between EPViz and existing tools

EPViz provides a streamlined interface that allows users to easily interact with the EEG data and visualize model predictions. Accordingly, EPViz fills an unoccupied niche amongst other EEG visualization and analyses tools. Perhaps the most widely used too is EDF Browser [57], which provides similar EDF loading and manipulation capabilities. EDF Browser also has a large suite of analytical tools, from filtering operations to spectral analysis [57]. While EDF Browser will remain a powerhouse in the EEG community, we believe that EPViz provides key functionality not incorporated in EDF Browser. First, EPViz is a streamlined and user-friendly application, which makes it easy for non-technical users to adopt. Second, it is geared towards spatio-temporal predictive analyses; the predictive overlay and topoplots are currently not supported in EDF Browser. Finally, it generates high-quality images of the data and results, which can be used in scientific publications and presentations.

There are also a variety of EEG software toolboxes developed for Matlab. For example, the popular EEGLab focuses on independent component analysis (ICA) and other time-frequency techniques [53, 54]. It also provides a GUI for visualizing events detected by these methods. In contrast, EEGNET provides tools for functional connectivity analysis [55] and includes a pipeline for the relevant preprocessing to construct an EEG connectome. Finally, BrainStorm registers multiple data formats including MEG, EEG, and MRI producing visualizations and analysis [56]. While these packages provide some overlapping functionality to EPViz, they do not lend themselves towards predictive analysis, like our PyTorch integration. They also rely on Matlab, which is an expensive and not-universally available platform. In contrast, EPViz is based on open-source Python packages and is freely available to the community. Table 2 summarizes the key features offered by EPViz, as compared to existing software packages. As seen, EPViz fills a much needed gap in EEG visualization.

**Table 2:** Summary of features provided by EPViz, as compared to existing EEG software.

	EPViz	EDF Browser	EEGLab	EEGNET	BrainStorm
View EEG Signals	✓	✓	✓	✓	✓
Compute Summary Statistics	✓	✓	✓	✓	✓
Annotate Data	✓	✓	✓		✓
PyTorch Integration	✓				
Overlay Predictions	✓				
Save High-Res Images	✓	✓			
License-Free	✓	✓			✓

Finally, EPViz leverages the MNE library [58] to produce topoplots in a user-friendly manner. While the native MNE library provides many visualization, preprocessing, and analysis tools, using them requires advanced scripting knowledge.

### Application domains

EPViz can be used in a variety of clinical and research applications, where the goal is to detect an event from the EEG data. One natural domain is epilepsy. In fact, the experimental testbed in this paper uses EPViz to compare the seizure detection performance across different machine learning methods. Other phenomenon of interest are auras and non-epileptic events, both of which can be detected using a similar training and evaluation strategy. Going one step further, EPViz supports channel-wise predictions, which makes it a natural tool for *seizure localization* studies [45, 46], where the goal is to identify a specific area of onset (e.g., lobe and/or hemisphere) and track the seizure activity as it propagates from that location.

Another application domain is BCI. Once again, some studies try to predict subject-level events, such as viewing a particular stimuli, while others zone in on specific EEG channels to tease apart the activity.

Finally, even though we have focused this paper on predictive analytics, the features of EPViz can be used to emphasize other aspects of the data. Recall that EPViz can overlay “predictions” contained in an auxiliary file. Hence, the user can create “predictions” that correspond to different experimental conditions. Another option is to create “predictions” that select certain EEG channels and time intervals based on an ERP analysis. Thus, EPViz is a flexible tool that users can adapt for their own needs.

### Limitations and future work

EPViz is currently designed to load and manipulate a single EEG recording. This setup makes it amenable to the *testing* phase of machine learning approaches, i.e., evaluating the performance of a model on new data. However, EPViz cannot be used for *model training*, which would require processing multiple EEG recordings at a time. Future work will integrate command-line options into EPViz for the user to *train* a PyTorch deep learning model given a data directory and subject ID list. This trained model can be loaded back into EPViz and applied to new EEG data using the existing functionality. Along the same lines, we will add an option for users to load multiple EEG recordings and toggle between them in the main display pane.

From a visualization standpoint, EPViz is optimized for the 10-20 electrode placement system [61]. While the user can load data from other montages, the signals will not be colored according to hemisphere, and EPViz will not generate a topoplot since the electrode placements are not provided in the EDF file. Future work will tackle this issue by allowing the user to add the electrode positions, hemisphere information, and desired ordering to the auxiliary text file mentioned above.

Along the same lines, EPViz has difficulty displaying more than 50 EEG signals at a time. Not only are the signals difficult to see, but the plot updates much more slowly after user interaction (e.g., signal filtering, scrolling through time, zoom functionality). To address this issue, we will integrate a memory management system that allows EPViz to efficiently cache and update data as needed. We will also add a pop-out window for the main display pane to accommodate the additional EEG signals.

Finally, EPViz is configured to apply the loaded models to the entire EEG recording to obtain predictions. While suitable for research purposes, clinicians desire real-time analysis capabilities to assist in their review of continuous recordings. We will explore such add-ons to EPViz in the future. These will likely rely on memory management systems and closer integration with existing clinical software packages.

## Conclusion

We have introduced EPViz, a lightweight and user-friendly visualizer for EEG data. EPViz is designed for predictive modeling applications, which are becoming increasingly popular in EEG research. Specifically, EPViz allows the user to generate and overlay predictions on top of the EEG signals, thus providing a mechanism to interpret the model output with respect to the data. EPViz can also generate high-quality images of the predictive modeling outputs to aid in scientific reporting [62]. EPViz is completely open-source and uses Python, which is the fastest-growing programming language for machine learning. Finally, EPViz has been designed for both engineers and clinician-scientists. In particular, we have included spectrogram visualization, which is often used in clinical review of EEG, and a built-in anonymizer to remove identifiable information from the EDF files. EPViz has helped our own team to build an interdisciplinary and inter-institutional collaboration in epilepsy. We hope that it will promote such collaborations for other researchers.

## References

[1] Marcuse LV, Fields MC, Yoo JJ. Rowan’s Primer of EEG E-Book. Elsevier Health Sciences; 2015.

[2] Krauss GL, Fisher RS. The Johns Hopkins atlas of digital EEG: an interactive training guide. Johns Hopkins University Press; 2006.

[3] Mitzdorf U. Current source-density method and application in cat cerebral cortex: investigation of evoked potentials and EEG phenomena. Physiological reviews. 1985;65(1):37–100. doi: 10.1152/physrev.1985.65.1.37 3880898

[4] Luck SJ, Kappenman ES. The Oxford handbook of event-related potential components. Oxford university press; 2011.

[5] Adeli H, Ghosh-Dastidar S, Dadmehr N. A wavelet-chaos methodology for analysis of EEGs and EEG subbands to detect seizure and epilepsy. IEEE Transactions on Biomedical Engineering. 2007;54(2):205–211. doi: 10.1109/TBME.2006.886855 17278577

[6] Bandarabadi M, Teixeira CA, Rasekhi J, Dourado A. Epileptic seizure prediction using relative spectral power features. Clinical Neurophysiology. 2015;126(2):237–248. doi: 10.1016/j.clinph.2014.05.022 24969376

[7] Logesparan L, Casson AJ, Rodriguez-Villegas E. Optimal features for online seizure detection. Medical & Biological Engineering & Computing. 2012;50(7):659–669. doi: 10.1007/s11517-012-0904-x 22476713

[8] Luckett P, Pavelescu E, McDonald T, Hively L, Ochoa J. Predicting state transitions in brain dynamics through spectral difference of phase-space graphs. Journal of computational neuroscience. 2019;46(1):91–106. doi: 10.1007/s10827-018-0700-1 30315514

[9] Li A, Chennuri B, Subramanian S, Yaffe R, Gliske S, Stacey W, et al. Using network analysis to localize the epileptogenic zone from invasive EEG recordings in intractable focal epilepsy. Network Neuroscience. 2018;2(02):218–240. doi: 10.1162/netn_a_00043 30215034 PMC6130438

[10] Micheloyannis S, Pachou E, Stam CJ, Vourkas M, Erimaki S, Tsirka V. Using graph theoretical analysis of multi channel EEG to evaluate the neural efficiency hypothesis. Neuroscience letters. 2006;402(3):273–277. doi: 10.1016/j.neulet.2006.04.006 16678344

[11] Onton J, Westerfield M, Townsend J, Makeig S. Imaging human EEG dynamics using independent component analysis. Neuroscience & biobehavioral reviews. 2006;30(6):808–822. doi: 10.1016/j.neubiorev.2006.06.007 16904745

[12] Alotaiby TN, Alshebeili SA, Alshawi T, Ahmad I, El-Samie FEA. EEG seizure detection and prediction algorithms: a survey. EURASIP Journal on Advances in Signal Processing. 2014;2014(1):183. doi: 10.1186/1687-6180-2014-183

[13] Orosco L. a survey of performance and techniques for automatic epilepsy detection. Journal of Medical and Biological Engineering. 2013;33(6):526–537. doi: 10.5405/jmbe.1463

[14] Osorio I, Zaveri HP, Frei MG, Arthurs S. Epilepsy: the intersection of neurosciences, biology, mathematics, engineering, and physics. CRC press; 2016.

[15] Alickovic E, Kevric J, Subasi A. Performance evaluation of empirical mode decomposition, discrete wavelet transform, and wavelet packed decomposition for automated epileptic seizure detection and prediction. Biomedical signal processing and control. 2018;39:94–102. doi: 10.1016/j.bspc.2017.07.022

[16] Faust O, Acharya UR, Adeli H, Adeli A. Wavelet-based EEG processing for computer-aided seizure detection and epilepsy diagnosis. Seizure. 2015;26:56–64. doi: 10.1016/j.seizure.2015.01.012 25799903

[17] Ghosh-Dastidar S, Adeli H, Dadmehr N. Mixed-band wavelet-chaos-neural network methodology for epilepsy and epileptic seizure detection. IEEE transactions on biomedical engineering. 2007;54(9):1545–1551. doi: 10.1109/TBME.2007.891945 17867346

[18] Kaleem M, Guergachi A, Krishnan S. Patient-specific seizure detection in long-term EEG using wavelet decomposition. Biomedical Signal Processing and Control. 2018;46:157–165. doi: 10.1016/j.bspc.2018.07.006

[19] Khan Y, Gotman J. Wavelet based automatic seizure detection in intracerebral electroencephalogram. Clinical Neurophysiology. 2003;114(5):898–908. doi: 10.1016/S1388-2457(03)00035-X 12738437

[20] Ocak H. Automatic detection of epileptic seizures in EEG using discrete wavelet transform and approximate entropy. Expert Systems with Applications. 2009;36(2):2027–2036. doi: 10.1016/j.eswa.2007.12.065

[21] Zandi AS, et al. Automated real-time epileptic seizure detection in scalp EEG recordings using an algorithm based on wavelet packet transform. IEEE Transactions on Biomedical Engineering. 2010;57(7):1639–1651. doi: 10.1109/TBME.2010.2046417 20659825

[22] Craley J, Johnson E, Venkataraman A. A Spatio-Temporal Model of Seizure Propagation in Focal Epilepsy. IEEE Transactions on Medical Imaging. 2019; p. 1–1. doi: 10.1109/TMI.2019.2950252 31675325

[23] Craley J, Johnson E, Venkataraman A. A novel method for epileptic seizure detection using coupled hidden markov models. In: International Conference on Medical Image Computing and Computer-Assisted Intervention. Springer; 2018. p. 482–489.

[24] Saab M, Gotman J. A system to detect the onset of epileptic seizures in scalp EEG. Clinical Neurophysiology. 2005;116(2):427–442. doi: 10.1016/j.clinph.2004.08.004 15661120

[25] Shoeb AH. Application of machine learning to epileptic seizure onset detection and treatment. Massachusetts Institute of Technology; 2009.

[26] Shoeb AH, Guttag JV. Application of machine learning to epileptic seizure detection. In: International Conference on Machine Learning; 2010. p. 975–982.

[27] Acharya UR, et al. Automated diagnosis of epileptic EEG using entropies. Biomedical Signal Processing and Control. 2012;7(4):401–408. doi: 10.1016/j.bspc.2011.07.007

[28] Andrzejak RG, Lehnertz K, Mormann F, Rieke C, David P, Elger CE. Indications of nonlinear deterministic and finite-dimensional structures in time series of brain electrical activity: Dependence on recording region and brain state. Physical Review E. 2001;64(6):061907. doi: 10.1103/PhysRevE.64.061907 11736210

[29] Esteller R, Echauz J, Tcheng T, Litt B, Pless B. Line length: an efficient feature for seizure onset detection. In: Engineering in Medicine and Biology Society, 2001. Proceedings of the 23rd Annual International Conference of the IEEE. vol. 2. IEEE; 2001. p. 1707–1710.

[30] Mormann F, Lehnertz K, David P, Elger CE. Mean Phase Coherence as a Measure for Phase Synchronization and its Application to the EEG of Epilepsy Patients. Physica D: Nonlinear Phenomena. 2000;144(3):358–369. doi: 10.1016/S0167-2789(00)00087-7

[31] Mormann F, Kreuz T, Andrzejak RG, David P, Lehnertz K, Elger CE. Epileptic Seizures are Preceded by a Decrease in Synchronization. Epilepsy Research. 2003;53(3):173–185. doi: 10.1016/S0920-1211(03)00002-0 12694925

[32] Craley J, Johnson E, Jouny C, Venkataraman A. Automated inter-patient seizure detection using multichannel Convolutional and Recurrent Neural Networks. Biomedical Signal Processing and Control. 2021;64:102360. doi: 10.1016/j.bspc.2020.102360

[33] Affes A, Mdhaffar A, Triki C, Jmaiel M, Freisleben B. A Convolutional Gated Recurrent Neural Network for Epileptic Seizure Prediction. In: International Conference on Smart Homes and Health Telematics. Springer; 2019. p. 85–96.

[34] Gao Y, Gao B, Chen Q, Liu J, Zhang Y. Deep Convolutional Neural Network-Based Epileptic Electroencephalogram (EEG) Signal Classification. Frontiers in Neurology. 2020;11. doi: 10.3389/fneur.2020.00375 32528398 PMC7257380

[35] Güler NF, et al. Recurrent neural networks employing Lyapunov exponents for EEG signals classification. Expert systems with applications. 2005;29(3):506–514. doi: 10.1016/j.eswa.2005.04.011

[36] Hu X, Yuan S, Xu F, Leng Y, Yuan K, Yuan Q. Scalp EEG classification using deep Bi-LSTM network for seizure detection. Computers in Biology and Medicine. 2020;124:103919. doi: 10.1016/j.compbiomed.2020.103919 32771673

[37] Khan H, Marcuse L, Fields M, Swann K, Yener B. Focal onset seizure prediction using convolutional networks. IEEE Transactions on Biomedical Engineering. 2017;65(9):2109–2118. doi: 10.1109/TBME.2017.2785401 29989952

[38] Oâ€™Shea A, Lightbody G, Boylan G, Temko A. Neonatal seizure detection from raw multi-channel EEG using a fully convolutional architecture. Neural Networks. 2020;123:12–25. doi: 10.1016/j.neunet.2019.11.023 31821947

[39] Park C, Choi G, Kim J, Kim S, Kim TJ, Min K, et al. Epileptic seizure detection for multi-channel EEG with deep convolutional neural network. In: 2018 International Conference on Electronics, Information, and Communication (ICEIC). IEEE; 2018. p. 1–5.

[40] Vidyaratne L, Glandon A, Alam M, Iftekharuddin KM. Deep recurrent neural network for seizure detection. In: 2016 International Joint Conference on Neural Networks (IJCNN). IEEE; 2016. p. 1202–1207.

[41] Wei Z, Zou J, Zhang J, Xu J. Automatic epileptic EEG detection using convolutional neural network with improvements in time-domain. Biomedical Signal Processing and Control. 2019;53:101551. doi: 10.1016/j.bspc.2019.04.028

[42] Yuan Y, Xun G, Jia K, Zhang A. A Multi-View Deep Learning Framework for EEG Seizure Detection. IEEE journal of biomedical and health informatics. 2018;23(1):83–94. doi: 10.1109/JBHI.2018.2871678 30624207

[43] Zhou M, Tian C, Cao R, Wang B, Niu Y, Hu T, et al. Epileptic seizure detection based on EEG signals and CNN. Frontiers in neuroinformatics. 2018;12:95. doi: 10.3389/fninf.2018.00095 30618700 PMC6295451

[44] Zou L, Liu X, Jiang A, Zhousp X. Epileptic Seizure Detection Using Deep Convolutional Network. In: 2018 IEEE 23rd International Conference on Digital Signal Processing (DSP). IEEE; 2018. p. 1–4.

[45] Craley J, Johnson E, Venkataraman A. Integrating convolutional neural networks and probabilistic graphical modeling for epileptic seizure detection in multichannel EEG. In: International Conference on Information Processing in Medical Imaging. Springer; 2019. p. 291–303.

[46] Craley J, Johnson E, Jouny C, Venkataraman A. Automated Noninvasive Seizure Detection and Localization Using Switching Markov Models and Convolutional Neural Networks. In: International Conference on Medical Image Computing and Computer-Assisted Intervention. Springer; 2019. p. 253–261.

[47] Abiri R, Borhani S, Sellers EW, Jiang Y, Zhao X. A comprehensive review of EEG-based brain–computer interface paradigms. Journal of neural engineering. 2019;16(1):011001. doi: 10.1088/1741-2552/aaf12e 30523919

[48] He B, Baxter B, Edelman BJ, Cline CC, Wenjing WY. Noninvasive brain-computer interfaces based on sensorimotor rhythms. Proceedings of the IEEE. 2015;103(6):907–925. doi: 10.1109/jproc.2015.2407272 34334804 PMC8323842

[49] Yuan H, He B. Brain–computer interfaces using sensorimotor rhythms: current state and future perspectives. IEEE Transactions on Biomedical Engineering. 2014;61(5):1425–1435. doi: 10.1109/TBME.2014.2312397 24759276 PMC4082720

[50] LaFleur K, Cassady K, Doud A, Shades K, Rogin E, He B. Quadcopter control in three-dimensional space using a noninvasive motor imagery-based brain–computer interface. Journal of neural engineering. 2013;10(4):046003. doi: 10.1088/1741-2560/10/4/046003 23735712 PMC3839680

[51] Vialatte FB, Maurice M, Dauwels J, Cichocki A. Steady-state visually evoked potentials: focus on essential paradigms and future perspectives. Progress in neurobiology. 2010;90(4):418–438. doi: 10.1016/j.pneurobio.2009.11.005 19963032

[52] Chen X, Wang Y, Nakanishi M, Gao X, Jung TP, Gao S. High-speed spelling with a noninvasive brain–computer interface. Proceedings of the national academy of sciences. 2015;112(44):E6058–E6067. doi: 10.1073/pnas.1508080112 26483479 PMC4640776

[53] Delorme A, Makeig S. EEGLAB: an open source toolbox for analysis of single-trial EEG dynamics including independent component analysis. Journal of neuroscience methods. 2004;134(1):9–21. doi: 10.1016/j.jneumeth.2003.10.009 15102499

[54] Delorme A, Mullen T, Kothe C, Akalin Acar Z, Bigdely-Shamlo N, Vankov A, et al. EEGLAB, SIFT, NFT, BCILAB, and ERICA: new tools for advanced EEG processing. Computational intelligence and neuroscience. 2011;2011. 10.1155/2011/130714 PMC3114412 21687590

[55] Hassan M, Shamas M, Khalil M, El Falou W, Wendling F. EEGNET: An open source tool for analyzing and visualizing M/EEG connectome. PloS one. 2015;10(9):e0138297. doi: 10.1371/journal.pone.0138297 26379232 PMC4574940

[56] Tadel F, Baillet S, Mosher JC, Pantazis D, Leahy RM. Brainstorm: a user-friendly application for MEG/EEG analysis. Computational intelligence and neuroscience. 2011;2011. doi: 10.1155/2011/879716 21584256 PMC3090754

[57] EDFbrowswer;. https://www.teuniz.net/edfbrowser/.

[58] Gramfort A, Luessi M, Larson E, Engemann DA, Strohmeier D, Brodbeck C, et al. MEG and EEG data analysis with MNE-Python. Frontiers in Neuroscience. 2013;7. doi: 10.3389/fnins.2013.00267 24431986 PMC3872725

[59] Goldberger AL, Amaral LA, Glass L, Hausdorff JM, Ivanov PC, Mark RG, et al. PhysioBank, PhysioToolkit, and PhysioNet: Components of a new research resource for complex physiologic signals. Circulation. 2000;101(23):215–220. doi: 10.1161/01.CIR.101.23.e215 10851218

[60] Obeid I, Picone P. The Temple University Hospital EEG Data corpus. Frontiers in Neuroscience. 2016;10:196. doi: 10.3389/fnins.2016.00196 27242402 PMC4865520

[61] Jurcak V, et al. 10/20, 10/10, and 10/5 systems revisited: their validity as relative head-surface-based positioning systems. Neuroimage. 2007;34(4):1600–1611. doi: 10.1016/j.neuroimage.2006.09.024 17207640

[62] Currey D, Hsu D, Ahmed R, Venkataraman A, Craley J. Cross-site Epileptic Seizure Detection Using Convolutional Neural Networks. In: CISS: Conference on Information Sciences and Systems; 2021. p. 1–6.

